# Adding a Dimension to the Dichotomy: Affective Processes Are Implicated in the Relationship Between Autistic and Schizotypal Traits

**DOI:** 10.3389/fpsyt.2020.00712

**Published:** 2020-07-24

**Authors:** Felicity V. Larson, Adam P. Wagner, Katharine Chisholm, Renate L. E. P. Reniers, Stephen J. Wood

**Affiliations:** ^1^ Department of Paediatric Psychology, Leicestershire Partnership NHS Trust, Leicester, United Kingdom; ^2^ Norwich Medical School, University of East Anglia, Norwich, United Kingdom; ^3^ National Institute for Health Research (NIHR) Applied Research Collaboration East of England (EoE), Cambridge, United Kingdom; ^4^ Department of Psychology, Aston University, Birmingham, United Kingdom; ^5^ Institute for Mental Health, University of Birmingham, Birmingham, United Kingdom; ^6^ Institute of Clinical Sciences, University of Birmingham, Birmingham, United Kingdom; ^7^ Orygen, Melbourne, VIC, Australia; ^8^ The Centre for Youth Mental Health, University of Melbourne, Melbourne, VIC, Australia

**Keywords:** autism, schizotypy, psychosis, affect, emotion, empathy, executive functioning

## Abstract

**Introduction:**

There is a recognized increase in vulnerability to psychosis in autistic people (AP). However, the construct of psychosis (particularly schizophrenia) contains several distinct factors, making understanding the relationship between autism and psychosis complex. Previous research has suggested that affective lability may be particularly related to psychotic experiences for AP who have experienced psychosis (AP-P). There is also a suggestion that psychosis might be a state of extreme (over)empathizing, perhaps related to emotional processes.

**Method:**

We recruited three groups: AP-P (N = 23), a group of AP who had not experienced psychosis (AP-NP; N = 59) and a neurotypical control group (NC, N = 41). Participants completed measures of autistic traits, schizotypal traits (as a proxy for psychosis-proneness), emotional processes, and perspective taking (as a proxy for the type of empathizing most theoretically likely to be linked to psychosis). As well as comparisons between groups, regression analyses were used to understand the influence of dependent variables on schizotypal traits.

**Results:**

We found that AP-P had significantly higher rates of schizotypy (positive and disorganized), as well as higher rates of emotional difficulties. Across all groups, affective lability had a positive and significant association with positive and disorganized schizotypal traits. Differences in perspective taking between groups were small and generally non-significant, particularly in adjusted comparisons; additionally, its impact on schizotypy was small and non-significant.

**Discussion:**

Our findings suggest that positive and disorganized schizotypy, in particular, have a relationship with affective lability. This, in turn, supports the idea of emotional processes as related to the development of schizotypal traits and psychosis across all individuals, regardless of autism diagnostic status. We found no evidence of empathy relating to any subscale of schizotypy, or the total schizotypy score. We contend that emotional processes should be considered in exploration of the relationship between autism and schizotypy in future. This may help to explain some of the findings of overlap between these constructs in previous research. Factors known to affect neurodevelopment of emotion systems such as history of early trauma, challenges during pregnancy and birth, and early childhood experiences of adversity during critical windows of development need further consideration in future research.

## Introduction

Autism spectrum disorders (ASDs) are life-long neurodevelopmental conditions affecting an individual’s perception of, and interaction with, the world (1). ASD refers to a number of heterogeneous ‘autisms’ (2), conditions that share core features of unusual perceptual abilities (3), social communication difficulties (4), and difficulties interpreting social cues (5) but differ subtly from individual to individual. Since the first definition of autism, and Bleuler’s description of what we would now label ‘negative symptoms’ of psychotic mental illness (6), there has been persistent debate about the relationship between experiences of autistic people (AP; individuals who meet diagnostic criteria for ASD^1^) and experiences of mental illness, particularly psychosis. This study is an attempt to further explore and clarify the relationship between these concepts, with reference to other relevant psychological processes.

Chisholm and colleagues (7) reviewed eight possible models of relationship between ASD and schizophrenia spectrums disorders (SSDs), and concluded that the evidence was strongest for four models: the increased vulnerability model (AP are more at risk of psychosis due to their ASD, but the conditions are separate); the diametrical model (ASD and psychosis are opposite ends of a continuum of overlapping constructs); associated liabilities model (factors that increase risk of one condition also increase risk of the other, but they remain separate); and the multiple overlapping etiologies model (some factors that lead to developing ASD also lead to developing psychosis, but others do not, leading to distinct but often similar or overlapping presentations). From the available evidence, the authors were not able to demonstrate that one model was clearly superior. They highlight that these models may not be mutually exclusive, and that there are likely to be subgroups for which one or other model may provide the greatest explanatory power. Thus, any research into an overlap between ASD and psychosis will be informed by, and influence, discussion of an explanatory model of that overlap.

In order to understand the relationship between ASD and psychosis, researchers have attempted to map ASD traits and psychotic traits into the same conceptual ‘space’. A personality construct called schizotypy has been used as a proxy for ‘psychosis-proneness’ (8). Schizotypy is characterized by magical thinking, strange experiences, social withdrawal, and other features, and can broadly be categorized into factors called positive, negative, or disorganized (9). Like ASD, it can be considered a spectrum that blends into ‘normality’—all people have schizotypal traits, but these are usually not clinically significant. In higher quantities, schizotypal traits might lead to a diagnosis of schizotypal personality disorder, a condition strongly linked to psychosis (10). This makes it perhaps easier to compare ASD (a collection of traits) to schizotypy (another set of traits), rather than psychosis (a state that changes over time and might at any time be considered present or absent). Research has found correlations between subscales of schizotypy and ASD. For example, there is a robust overlap between negative symptoms of schizotypy and autistic traits in adolescents with ASD (11). Social skill deficit seems specific to ASD, and positive schizotypy (for example, unusual experiences such as believing in magic or psychic phenomena), seems specific to schizotypy in high functioning adults (12). Executive functioning processes have been implicated as a causal neurobiological mechanism that might explain both (13).

Using factor analysis, two of the largest studies in this area drew differing conclusions about the relationship between autistic and schizotypal traits. Dinsdale et al. (14) favored a two-factor solution, and argued that there was a clear division between autistic and schizotypal traits, adding further support for a theory that defined ASD and schizophrenia as diametrical opposites (15). Ford and Crewther (16), however, defined a three-factor solution that presents a more complex relationship between the traits. While there were two factors that segregated between the measures, indicating separate autistic (‘social disorganization’) and schizotypal (‘perceptual oddities’) constructs, these explained much less variance than the third factor which included both autistic and schizotypal traits. They term the construct that this factor measures ‘social rigidity’ and postulate that this factor underlies many of the difficulties experienced by both AP and people who experience high levels of schizotypy. Other evidence from research on those dually-diagnosed with ASD and psychosis indicates high rates of major mood disorders such as schizoaffective disorder or bi-polar disorder (17). This finding is also supported by genetic studies (18) and prevalence data (19) showing higher rates of bi-polar disorder in AP.

A factor that may be involved in the relationship between schizotypal and autistic traits is empathy. Empathy is a complex skill that involves predicting and reacting to how you believe another person will feel. It has been conceptualized as having broadly two factors: cognitive empathy (broadly, this is defined as understanding other people’s perspectives); and affective empathy (colloquially, feeling for another person). The diametric model of ASD and schizophrenia suggests that increased empathy may be linked to increased risk of psychosis, through a mechanism of overly empathizing with the perceived contents of others’ minds (15, 20). Empathy is found to be impaired in AP in general (21), and has been linked to Theory of Mind deficits, which have a strong basis in both the mirror neuron and executive functioning systems of the brain (22). Harmesen (23) has highlighted that both AP, those with a diagnosis of BPD and those with a diagnosis of bipolar disorder all show impaired cognitive empathy. Conversely, individuals with schizophrenia show impaired affective empathy. However, one particular subtype of empathy, perspective taking, has been found to be impaired in both AP (24) and, separately, in individuals with schizophrenia (25). Previous research has shown that there are differences in empathizing between AP-P and AP-NP (26), but cognitive and affective empathizing have not been considered separately in this population. It might reasonably be predicted that perspective taking may be differentially experienced by AP-P and AP-NP, on the basis of the above research, and further that it could play a role in our understanding of autistic and schizotypal traits.

In order to contribute to better understanding of this area, and the potential interactions between emotion regulation difficulties, affective lability, schizotypy, and autistic traits, we have attempted to investigate these concepts in the same theoretical space. The following hypotheses were tested:

H1. AP-P will use less effective emotion regulation strategies and report more affective lability than AP-NP
H2. AP-P will be better at perspective taking than AP-NP
H3. Schizotypal traits will be higher in AP with a history of psychosis (AP-P) compared with AP who have no history of psychosis (AP-NP)
H4. Emotion regulation difficulties and affective lability will be associated with higher schizotypal scores across participant groups

## Materials and Methods

Ethical approval for the study was given by the North of Scotland NHS Research Ethics Committee in January 2016. The study was conducted between January 2016 and April 2017.

### Design

An observational study comparing self-reported measures of autistic traits, schizotypal traits, and emotional processes between participant groups. Participants were recruited either *via* participation in previous research or *via* social media advertising, and were incentivized with the opportunity to participate in a prize draw.

### Participants

Participants were all adults (aged 18 or older), and were required to have English as their first language. They were recruited to three groups.

#### Autistic People With No Psychosis (AP-NP)

Recruited *via* the Autism Research Centre’s (ARC’s) database (https://www.autismresearchcentre.net/), they were asked to confirm they had no significant mental health history. ASD diagnosis was not confirmed, but the database is maintained by a respected research group who check participant eligibility: thus, we considered this group representative of AP. A total of 59 participants were recruited.

#### Autistic People with Psychosis (AP-P)

Consisted of:

- participants invited from previous research (17);
- new participants self-identified through the ARC database.

Participants from the ARC database were screened using the Diagnostic Interview for Psychosis (DIP-DM) (27), which generates diagnoses using the OPCRIT algorithm (28). Individuals meeting criteria for an SSD in DSM-IV-TR (29), ICD-10 (30), or Research Diagnostic Criteria (RDC) (31) systems were considered to have a confirmed history of psychosis. This replicates methods used in Larson et al. (17), and full details are given in Supplemental Materials.

A total of 23 participants were recruited from both sources.

#### Neurotypical Controls (NC)

Participants were recruited through social media advertising. Participants were not formally screened, but were asked to confirm they had neither history of ASD diagnosis nor any significant mental health history. A total of 41 participants were recruited.

### Measures

The following self-report measures were used:

- *Autism Spectrum Quotient (AQ)* (32): A 50-item questionnaire that measures traits associated with ASD. It contains five subscales (Communication, Social, Imagination, Local Details, and Attention Switching). The minimum score is zero, indicating low ASD traits, and the maximum is 50. A cut-off of 32 is considered indicative of probable ASD. The AQ has been shown to have good reliability and construct validity by the original authors and in later studies (33, 34), although subscale definitions have been questioned. Thus, we used the ‘total AQ’ summary score.
- *Schizotypal Personality Questionnaire—Brief Revised (SPQ-BR)* (35): A 32-item questionnaire measuring schizotypal traits. Contains nine subscales that can be categorized into three or four superordinate subscales: cognitive perceptual differences, interpersonal difference, and disorganized traits. SPQ-BR scores range from 32 (low overall schizotypy) to 160. Cognitive Perceptual (Positive Schizotypy) scores range from 14 to 70. Interpersonal (Negative Schizotypy) ranges from 10 to 50, and Disorganized ranges from eight to 40. It has been suggested that the Interpersonal subscale could be divided into two, separating out social anxiety (36); however, in line with previous studies we are seeking to replicate, we do not do so. The SPQ-BR as a whole has been shown to have reasonable reliability and validity in a large normative sample (36).
- *Affective Lability Scale-18 (ALS-18)* (37): This 18-item assesses the extent to which individuals switch between emotional states and comprises three subscales (anxiety/depression, depression/elation, and anger) assessing shifts between different emotional states. Higher scores indicate higher levels of affective lability, ranging from zero to 54. The anxiety/depression and anger subscales, each consisting of five items, have scores ranging from zero to 15. The depression/elation subscale consists of eight items and produces scores ranging from zero to 24. The measure has good psychometric properties (38).
- *Emotion Regulation Questionnaire-9 (ERQ-9)* (39): This is a nine item measure of the extent to which individuals utilize one of two distinct coping strategies to manage strong emotion: reappraisal and suppression. Results are given in relation to these two strategies. Individuals rate themselves from one to seven on each item, and some items are reverse-scored. Reappraisal is measured by five items, giving a score of five to 35. Suppression is measured by four items, giving a score of four to 20. Higher scores indicate higher levels of emotion regulation strategy use, and can be considered separately or as a total. The measure has good reliability and validity in community samples (40), and has improved psychometric properties compared to a previous, longer, version.
- Questionnaire of Cognitive and Affective Empathy (QCAE) (41), perspective-taking subscale: A 10-item subscale of a larger measure, it captures the cognitive empathy element related to the ability to imagine alternative perspectives other than one’s own. Higher scores indicate higher levels of alternative perspective-taking ability, ranging from zero to 10. It has good reliability and construct validity in a student sample (41).

All participants were asked their age and gender. No other demographics were collected.

### Analysis

Data were analyzed using R (R Core Team).

#### Descriptive Statistics and Univariate Comparisons

Initially, the AP-NP, AP-P and NC groups were compared in terms of gender, age and the measures introduced in *Measures* section. Univariate comparisons between AP-NP and AP-P were made using Fisher’s Exact test and t-tests. Univariate comparisons across all three groups were made using Fisher’s Exact test and one-way analysis of variance (ANOVA). Non-parametric versions (Wilcoxon and Kruskal–Wallis rank-sum tests) of these comparisons, along with appropriate descriptive statistics, are reported in the Supplementary Materials. These sets of analyses are compared to determine robustness of conclusions.

#### Group Comparisons of ALS-18, ERQ-9 and QCAE Perspective Taking

To begin examining our hypotheses, unadjusted comparisons of affective and empathy measures between groups were conducted as outlined in *Descriptive Statistics and Univariate Comparisons* section. Adjusted group comparisons were made using linear regression, with a separate regression for each scale. Independent variables (excluding the outcome scale in the corresponding regression) included: Participant Group; Gender; Age; ALS-18 Total; ERQ-9 Cognition Reappraisal; ERQ-9 Emotion Suppression; QCAE Perspective Taking.

#### Schizotypy Regressions

Linear regression models were used to look for adjusted relationships with SPQ-BR scales (Total and all subscales). In these models, the following independent variables were included: Participant Group; Gender; Age; AQ Total; ALS-18 Total; ERQ-9 Cognition Reappraisal; ERQ-9 Emotion Suppression; QCAE Perspective Taking. In each regression, multicollinearity was examined using variation inflation factors (VIFs).

For each regression, a key area of interest was the potential interaction between Participant Group and each of the dependent variables. To explore these interactions, we fitted a series of models that extended each base regression with all possible combinations of (first order) interactions between Group and other dependent variables. Fitted models were compared using the second-order Akaike Information Criterion (AICc) (42, 43), where lower values indicate a better fitting model. The AICc, as opposed to the standard AIC, was used given the sample size. We considered models with AICcs within two of the lowest AICc (details given in the Supplementary materials), as models within this range are not considered distinguishable. This allowed us to check the robustness of the fit of the best model. Where this set of models included the model with no interactions, we choose this model for greater parsimony. Residual plots of the finally selected models were reviewed to check model fit.

Where interactions were fitted, the Effects (44) package in R was used to plot their effect.

#### Secondary Analysis: Exploring ALS-18 Subscales

Having conducted the analysis outlined in the above sections, affective lability was highlighted as a key factor. Thus, we repeated the analyses described in *Descriptive Statistics and Univariate Comparisons*, *Group Comparisons of ALS-18, ERQ-9 and QCAE Perspective Taking* and *Schizotypy Regressions* sections, replacing ALS-18 Total with its subscales.

#### Effect Size Measures

Pearson’s correlation coefficient (r) was used as the primary measure of effect size. Generally, Pearson’s r was calculated from t-statistics (45), using a formula from Rosenthal (46). For the one-way ANOVA, eta-squared effect measures were converted to r using formulas from Cohen (47) and Rosenthal (46)—the common effect size measure easing comparison. Effect sizes for r were considered small (<0.1), medium (<0.3) and large (<0.5) (48). To further assist interpretation, regression models fit in the main paper were refitted in the **Supplementary Materials** with their continuous variables standardized to zero mean and unit variance.

## Results

### Participant Group Size, Descriptive Statistics and Univariate Comparisons

Group sizes, descriptive statistics and tests of differences between groups (including confidence intervals, p-values and effect sizes) are reported in **Table 1** and non-parametric forms of these comparisons are reported in **Table S1**.

**Table 1:** Descriptive statistics.

	**AP-NP**		**AP-P**		**NC**		**ASD comparison (AP-NP v AP-P)**						**All group comparison**		
	**Mean**	**SD**	**Mean**	**SD**	**Mean**	**SD**	**Stats comp**	**OR/diff**	**LCI**	**UCI**	**P-value**	**r**	**Stats comp**	**P-value**	**r**	
N=	59		23		41											
Gender (% W)	56%		26%		61%		Fisher’s	0.28	0.08	0.88	***0.026***	0.33	Fisher’s	***0.020***	–	
Age (years)	44.3	12.8	33.5	11.0	31.5	9.4	t(47) = 3.8	10.9	5.1	16.6	***<0.001***	0.49	F(2,120) = 17.4	***<0.001***	0.47	
AQ Total	39.0	8.2	31.0	9.9	19.4	9.1	t(35) = 3.4	8.0	3.3	12.7	***0.002***	0.51	F(2,120) = 59.3	***<0.001***	0.71	
SPQ-BR Total	71.8	16.6	77.6	21.7	52.3	20.0	t(33) = −1.2	−5.8	−16.0	4.4	0.256	0.20	F(2,120) = 18	***<0.001***	0.48	
SPQ-BR Positive	20.6	9.2	29.1	13.4	15.2	9.1	t(30) = −2.8	−8.5	−14.7	−2.3	***0.009***	0.45	F(2,120) = 14.1	***<0.001***	0.44	
SPQ-BR Negative	28.4	7.6	26.8	8.1	19.4	8.9	t(38) = 0.8	1.6	−2.3	5.6	0.414	0.13	F(2,120) = 15.6	***<0.001***	0.46	
SPQ-BR Disorganized	22.7	6.1	21.7	4.7	17.8	7.3	t(51) = 0.9	1.1	−1.5	3.6	0.398	0.12	F(2,120) = 7.6	***<0.001***	0.34	
ALS-18 Total	19.2	13.3	26.0	10.8	17.8	12.8	t(49) = −2.4	−6.8	−12.5	−1.1	***0.021***	0.32	F(2,120) = 3.3	***0.041***	0.23	
ALS-18 Anxiety/Depression	6.3	4.3	8.3	4.3	5.9	5.0	t(40) = −1.9	−2.0	−4.2	0.1	0.065	0.29	F(2,120) = 2.3	0.104	0.19	
ALS-18 Depression/Elation	8.9	6.6	11.9	4.9	9.0	6.8	t(54) = −2.2	−3.0	−5.6	−0.3	***0.032***	0.29	F(2,120) = 2	0.142	0.18	
ALS-18 Anger	3.9	4.2	5.7	4.2	3.0	3.5	t(40) = −1.8	−1.8	−3.9	0.3	0.088	0.27	F(2,120) = 3.6	***0.031***	0.24	
ERQ-9 Cognitive Reappraisal	22.3	7.0	19.7	6.4	22.0	6.4	t(44) = 1.6	2.6	−0.6	5.9	0.110	0.24	F(2,120) = 1.3	0.270	0.15	
ERQ-9 Emotion Suppression	16.3	6.4	17.3	5.9	14.5	5.7	t(43) = −0.7	−1.0	−4.0	2.0	0.500	0.10	F(2,120) = 1.8	0.171	0.17	
QCAE Perspective Taking	20.3	7.0	22.2	6.7	29.2	5.8	t(42) = −1.2	−2.0	−5.3	1.4	0.246	0.18	F(2,120) = 23.1	***<0.001***	0.53	

Means, standard deviation (SD) by participant group and comparisons between (i) AP groups (Fisher’s exact test/two-sample t-test) and (ii) all three groups (Fisher’s exact test/ANOVA).

Bold/italic p-values indicate significant differences at the 5% level.

AP-NP, Autistic people with no psychosis; AP-P, Autistic people with psychosis; NC, Neurotypical controls; Stats. Comp, Statistical comparison; OR/diff, Odds ratio/difference; LCI/UCI, Lower/upper 95% confidence interval; W, Women; SPQ-BR, Schizotypal Personality Questionnaire; ALS-18, Affective lability scale-18; ERQ-9, Emotional regulation questionnaire-9; QCAE, Questionnaire of cognitive and affective lability. There are two separate sets of comparisons: the first compares the AP groups (middle set of columns) and the second compares all three groups (last set of columns).

Gender split was similar between AP-NP and NC groups; however, there were significantly (proportionately) fewer women in AP-P compared to AP-NP. The AP-NP group was significantly older than the AP-P and NC groups. A large and significant difference in AQ scores was found between AP-NP and AP-P. As would be expected, the NC group had the lowest AQ score. Between AP-NP and AP-P groups, across SPQ-BR scales, the only significant difference was found on the Positive scale. NC had the lowest means across all SPQ-BR scales. The same differences between groups were found in the equivalent non-parametric analysis reported in **Table S1**.

### Group Comparisons of ALS-18, ERQ-9 and QCAE Perspective Taking Scores

Detailed unadjusted comparisons of these measures are given in **Table 1** (including test statistics, 95% confidence intervals (CIs), p-values and effect sizes). Between AP-NP and AP-P, there were only significant unadjusted differences on ALS-18 Total. The NC group only significantly differed to the AP-NP and AP-P groups on QCAE Perspective Taking.

Detailed adjusted comparisons of these measures are given in **Table 2** (including 95% CIs, p-values and effect sizes; equivalent standardized versions are given in **Table S2**). The only significant adjusted differences included: the NC group scoring significantly higher than AP-NP group on ALS-18 Total; and NC had significantly higher QCAE Perspective Taking scores than both AP-NP and AP-P.

**Table 2:** Fitted regressions on ALS-18 Total, ERQ-9 and QCAE scales.

Variable	Cat. level	Adjusted comparison regressions (n = 123)																			
		ALS-18 Total (adjusted R² = 0.43)					ERQ-9 Cognitive Reappraisal (adjusted R² = 0.08)					ERQ-9 Emotion Suppression (adjusted R² = 0.43)					QCAE Perspective Taking(adjusted R² = 0.67)				
		b	95% CI		P-value	r	B	95% CI		P-value	r	b	95% CI		P-value	r	b	95% CI		P-value	r
Intercept	–	−6.0	−26.7	14.7	0.567	0.054	11.6	−1.8	25.0	0.090	0.160	4.7	−5.0	14.4	0.343	0.090	37.0	30.8	43.3	***<0.001***	0.743
Participant Group*	AP-P	3.4	−2.6	9.3	0.266	0.105	−2.1	−6.0	1.8	0.289	0.101	0.7	−2.1	3.5	0.626	0.046	−2.3	−5.0	0.3	0.086	0.162
	NC	7.1	1.1	13.1	***0.020***	0.218	−0.7	−4.7	3.3	0.723	0.034	2.4	−0.5	5.2	0.105	0.153	−1.4	-4.1	1.4	0.330	0.092
Gender^†^	W	0.9	−3.0	4.9	0.636	0.045	0.1	−2.5	2.7	0.945	0.007	−2.4	−4.2	−0.6	***0.008***	0.247	0.8	−1.0	2.5	0.392	0.081
Age (years)	–	−0.2	−0.3	0.0	***0.023***	0.213	0.0	−0.1	0.1	0.534	0.059	0.0	−0.1	0.1	0.659	0.042	0.0	-0.1	0.1	0.984	0.002
AQ Total	–	0.4	0.1	0.7	***0.025***	0.211	0.1	−0.1	0.3	0.484	0.067	0.0	−0.1	0.2	0.683	0.039	−0.5	−0.6	−0.3	***<0.001***	0.569
SPQ-BR Positive	–	0.5	0.3	0.7	***<0.001***	0.406	0.1	0.0	0.3	0.122	0.146	0.0	−0.1	0.1	0.411	0.078	0.1	0.0	0.2	0.243	0.111
SPQ-BR Negative	–	0.0	−0.3	0.4	0.826	0.021	0.0	−0.2	0.2	0.953	0.006	0.5	0.3	0.6	***<0.001***	0.503	−0.1	−0.3	0.0	0.122	0.146
SPQ-BR Disorganized	–	0.4	0.1	0.7	***0.012***	0.236	0.1	−0.2	0.3	0.632	0.045	−0.2	−0.3	0.0	***0.039***	0.195	−0.1	−0.2	0.1	0.508	0.063
ALS-18 Total	–						−0.1	−0.3	0.0	***0.022***	0.215	0.0	−0.1	0.1	0.894	0.013	0.0	0.0	0.1	0.354	0.088
ERQ-9 Cognitive Reappraisal	–	−0.3	−0.6	0.0	***0.022***	0.215						0.0	−0.2	0.1	0.580	0.053	0.2	0.0	0.3	***0.017***	0.224
ERQ-9 Emotion Suppression	–	0.0	−0.4	0.4	0.894	0.013	−0.1	−0.3	0.2	0.580	0.053						0.1	−0.1	0.2	0.554	0.056
QCAE Perspective Taking	–	0.2	−0.2	0.6	0.354	0.088	0.3	0.1	0.6	***0.017***	0.224	0.1	−0.1	0.3	0.554	0.056					

Bold/italic p-values indicate significant terms at the 5% level. Gray cells indicate that the corresponding term has not been included in the regression. Cat., Categorical; SPQ-BR, Schizotypal Personality Questionnaire; CI, Confidence interval; AP-P, Autistic people with psychosis; NC, Neurotypical controls; W, Woman; AQ, Autism quotient; ALS-18, Affective lability scale-18; ERQ-9, Emotional regulation questionnaire-9; QCAE, Questionnaire of cognitive and affective lability. *‘AP-NP’ group taken as reference level. ^†^‘Man’ taken as reference level.

### Schizotypy Regressions: Controlled Relationships With Schizotypy Scores

Regression models within two of the best AICc for each SPQ-BR scale (Total, Positive, Negative and Disorganized) are shown in **Table S3**. For three of the models (Total, Positive and Disorganized) the model including no interactions with Group gave the best fitting model (highlighted in yellow in **Table S1**); additionally, there were no consistently included interactions across the other models considered. For the Negative scale, the model with no interactions did *not* have the lowest AICc; however, its fit is only negligibly worse than the best fitting model (765.52 versus 764.38)—given this fit, and the lack of consistent interactions included across the other models, we chose the model with no interactions for reasons of parsimony. Residual plots indicate no substantial problems with model fit. Except for one variable, all VIFs are below four; the remaining variable has a VIF of just over five.

The fit of the final models is given in **Table 3** (including 95% CIs, p-values and effect sizes; the resulting fit with continuous variables standardized is given in **Table S4**). The amount of variation explained varied from its lowest in disorganized schizotypy to highest in negative schizotypy. In each of the four regression models, the test of overall regression is significant (p-values <0.001).

**Table 3:** Fitted Schizotypal regressions.

Variable	Cat. level	SPQ-BR regressions (n = 123)																			
		Total (adjusted R² = 0.61)					Positive (adjusted R² = 0.41)					Negative (adjusted R² = 0.68)					Disorganized (adjusted R² = 0.28)				
		b	95% CI		P-value	r	b	95% CI		P-value	r	b	95% CI		P-value	r	b	95% CI		P-value	r
Intercept	–	18.9	−8.0	45.8	0.167	0.130	−4.2	−21.5	13.0	0.629	0.045	7.7	−2.7	18.2	0.144	0.137	15.4	4.0	26.8	0.009	0.244
Participant Group*	AP-P	6.2	−1.7	14.0	0.122	0.145	6.7	1.6	11.7	***0.010***	0.240	0.2	−2.8	3.2	0.895	0.012	−0.7	−4.0	2.6	0.673	0.040
	NC	−4.7	−12.9	3.6	0.265	0.105	−3.6	−8.9	1.7	0.178	0.126	−0.1	−3.3	3.1	0.946	0.006	−0.9	−4.4	2.5	0.595	0.050
Gender^†^	Woman	−1.1	−6.4	4.2	0.672	0.040	−0.5	−3.8	2.9	0.790	0.025	0.0	−2.0	2.1	0.979	0.002	−0.7	−2.9	1.5	0.535	0.058
Age (years)	–	0.1	−0.2	0.3	0.545	0.057	0.1	−0.1	0.2	0.356	0.087	0.0	−0.1	0.1	0.740	0.031	0.0	−0.1	0.1	0.740	0.031
AQ Total	–	0.5	0.1	1.0	***0.018***	0.221	0.0	−0.2	0.3	0.728	0.033	0.3	0.2	0.5	***<0.001***	0.345	0.2	0.0	0.3	0.115	0.148
ALS-18 Total	–	0.7	0.5	0.9	***<0.001***	0.505	0.4	0.3	0.5	***<0.001***	0.484	0.1	0.0	0.2	0.058	0.177	0.2	0.1	0.3	***<0.001***	0.354
ERQ-9 Cognitive Reappraisal	–	0.3	−0.1	0.7	0.118	0.147	0.2	0.0	0.5	0.090	0.159	0.0	−0.1	0.2	0.706	0.036	0.1	−0.1	0.2	0.432	0.074
ERQ-9 Emotion Suppression	–	0.7	0.3	1.2	***0.002***	0.291	0.3	0.0	0.6	***0.045***	0.187	0.6	0.4	0.7	***<0.001***	0.517	−0.1	−0.3	0.1	0.198	0.121
QCAE Perspective Taking	–	−0.1	−0.7	0.4	0.691	0.037	0.1	−0.2	0.5	0.453	0.071	−0.2	−0.4	0.1	0.143	0.137	−0.1	−0.3	0.1	0.465	0.069

Bold/italic p-values indicate significant terms at the 5% level. Cat., Categorical; SPQ-BR, Schizotypal Personality Questionnaire; CI, Confidence interval; AP-P, Autistic people with psychosis; NC, Neurotypical controls; AQ, Autism quotient; ALS-18, Affective lability scale-18; ERQ-9, Emotional regulation questionnaire-9; QCAE, Questionnaire of cognitive and affective lability. *’AP-NP’ group taken as reference level. ^†^’Man’ taken as reference level.

Between groups, schizotypy differences are minimal. For total schizotypy, there was a significant difference between NC and AP-P, with AP-P scoring approximately 11 units higher. On positive schizotypy, AP-P scored significantly higher than both AP-NP and NC. Other group differences were small and non-significant.

Across Positive, Negative and Disorganized scales, AQTotal was only significantly associated with negative schizotypy. This was the second largest effect on the Negative scale, and likely drives the significant relationship with AQTotal on the Total scale.

ALS-18 Total had a significant and the largest impact within each of the Positive and Disorganized scales. Accordingly, ALS-total significantly impacts on SPQ-BR Total and has the largest impact thereon. ERQ-9 Emotion Suppression had a significant effect on the Positive and Negative scales, and had the third and largest impact on these respectively. These relationships likely drive the significant impact of ERQ-9 Emotion Suppression on SPQ-BR Total.

There were no significant relationships between the SPQ-BR subscales and gender, age, ERQ-9 Cognitive Reappraisal, or QCAE Perspective Taking respectively.

With no interactions with Participant Group, we had no evidence for effects noted above differing by Participant Group (however, this is *not* proof for the contrary).

### Secondary Analysis: Exploration of ALS-18 Subscales

Unadjusted comparisons between groups on the three ALS-18 subscales are detailed in **Table 1**. AP-P scores higher on all three subscales, but only significantly so on the ALS-18 Depression/Elation scale. Differences between NC and AP-P are greatest, but non-significant; differences between NC and AP-NP are smaller.

Adjusted comparisons between groups on the three ALS-18 subscales are detailed in **Table S4** (and standardized in **Table S5**). In the adjusted comparisons, all group differences were non-significant and small, including direct comparisons between NC and AP-P groups.

**Figure 1:**
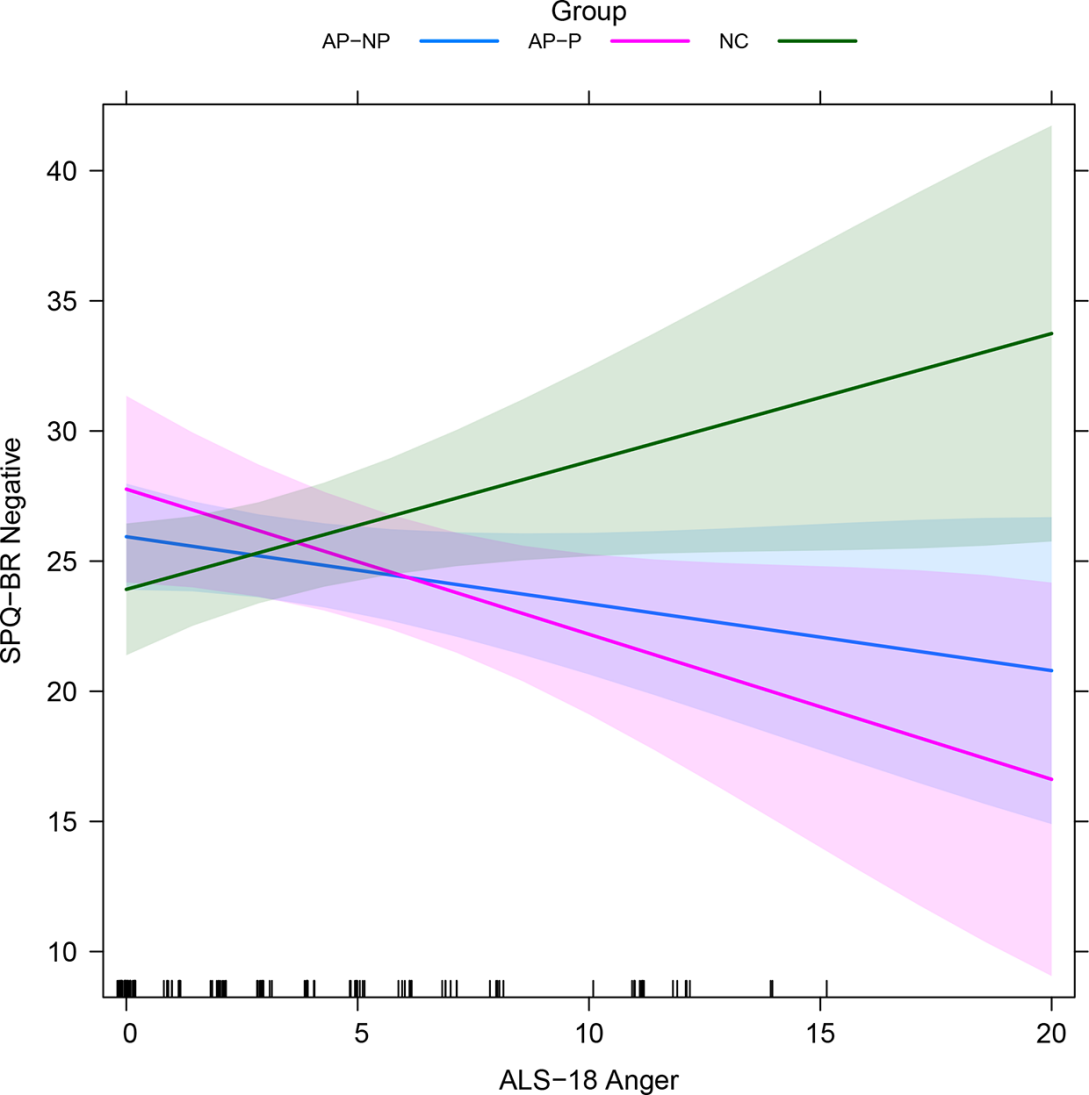
Fitted interactions between participant group and ALS-18 Anger in SPQ-BR Negative regression with ALS-18 subscales (see *Secondary Analysis: Exploration of ALS-18 Subscales*; averaged across other variables). Colored regions indicate the 95% confidence intervals. AP-NP, Autistic people with *no* psychosis; AP-P, Autistic people *with* psychosis; NC, Neurotypical controls; SPQ-BR, Schizotypal Personality Questionnaire; ALS-18, Affective lability scale-18.

Regressions relating the ALS-18 subscales to SPQ-BR measures, alongside other variables, are reported in **Table 4** (standardized version given in **Table S8**). A detailed report on these regressions is given in the Supplementary Results. Results were broadly similar to those reported in *Schizotypy Regressions: Controlled Relationships with Schizotypy Scores*. ALS-18 Anxiety/Depression significantly impacted on positive and negative schizotypy. ALS-18 Depression/Elation significantly impacted on disorganized schizotypy. These relationships likely drive the relationships between the total schizotypy and both of Anxiety/Depression and Depression/Elation. ALS-18 Anger generally does not impact on schizotypy scales except for negative schizotypy, where there is an interaction with participant group, as depicted in **Figure 1**: increases on ALS-18 Anger is associated with *increases* on negative schizotypy for NCs, but *decreases* for the AP-NP and AP-P groups. There was also interaction between participant group and ERQ-9 Cognitive reappraisal, as depicted in **Figure S1**: increases on cognitive reappraisal are associated with increases for NC, decreases for AP-P and very little change for AP-NP.

**Table 4:** Fitted Schizotypal regressions with ALS-18 subscales.

Variable	Cat. level	SPQ-BR regressions (with ALS-18 subscales) (n = 123)																				
		Total (adjusted R² = 0.62)					Positive (adjusted R² = 0.41)					Negative (adjusted R² = 0.74)					Disorganized (adjusted R² = 0.32)					
		b	95% CI		P-value	r	b	95% CI		P-value	r	b	95% CI		P-value	r	b	95% CI		P-value	r
Intercept	–	16.5	−11.2	44.2	0.239	0.112	−3.1	−21.0	14.9	0.734	0.032	5.3	−6.2	16.9	0.359	0.089	10.9	−0.6	22.4	0.063	0.175
Participant Group*	AP-P	6.1	−1.8	13.9	0.131	0.143	6.3	1.2	11.4	***0.016***	0.226	6.0	−3.1	15.1	0.196	0.125	0.0	−3.2	3.3	0.978	0.003
	NC	−4.9	−13.1	3.4	0.242	0.111	−4.0	−9.3	1.4	0.145	0.138	−9.9	−17.4	−2.5	***0.009***	0.248	−0.3	−3.8	3.1	0.851	0.018
Gender^†^	Woman	−2.6	−8.1	2.9	0.348	0.089	−0.9	−4.4	2.6	0.619	0.047	−0.9	−2.8	1.1	0.380	0.085	−0.9	−3.2	1.4	0.441	0.073
Age (years)	–	0.1	−0.1	0.3	0.476	0.068	0.1	−0.1	0.2	0.401	0.080	0.0	−0.1	0.1	0.900	0.012	0.0	−0.1	0.1	0.425	0.076
AQ total	–	0.6	0.1	1.0	***0.017***	0.225	0.0	−0.3	0.3	0.857	0.017	0.4	0.2	0.6	***<0.001***	0.400	0.2	0.0	0.4	***0.024***	0.212
ALS-18 Anxiety/Depression	–	1.3	0.5	2.1	***0.002***	0.295	0.7	0.2	1.2	***0.009***	0.245	0.6	0.3	0.8	***<0.001***	0.364	0.1	−0.3	0.4	0.726	0.033
ALS-18 Depression/Elation	–	0.6	0.1	1.2	***0.018***	0.223	0.3	−0.1	0.6	0.107	0.153	−0.1	−0.3	0.1	0.210	0.121	0.5	0.2	0.7	***<0.001***	0.364
ALS-18 Anger	–	0.0	−0.7	0.8	0.919	0.010	0.3	−0.2	0.9	0.184	0.126	−0.3	−0.6	0.1	0.145	0.140	−0.2	−0.5	0.2	0.302	0.098
ERQ-9 Cognitive Reappraisal	–	0.3	−0.1	0.7	0.089	0.161	0.2	0.0	0.5	0.082	0.164	0.0	−0.2	0.2	0.794	0.025	0.1	−0.1	0.2	0.392	0.081
ERQ-9 Emotion Suppression	–	0.7	0.3	1.2	***0.002***	0.282	0.3	0.0	0.6	***0.046***	0.188	0.6	0.4	0.7	***<0.001***	0.540	−0.2	−0.3	0.0	0.105	0.153
QCAE Perspective Taking	–	−0.1	−0.6	0.5	0.820	0.022	0.1	−0.2	0.5	0.492	0.065	−0.1	−0.3	0.1	0.441	0.075	0.0	−0.3	0.2	0.811	0.023
Participant Group × ALS-18 Anger	AP-P:Anger											−0.3	−0.9	0.3	0.304	0.099					
	NC : Anger											0.7	0.2	1.3	***0.006***	0.263					
Participant Group × ERQ-9 Cog. Reapp.	AP-P:ERQ-9 CR											−0.2	−0.6	0.2	0.338	0.093					
	NC : ERQ-9 CR											0.4	0.1	0.7	***0.018***	0.226					

Bold/italic p-values indicate significant terms at the 5% level. Cat., Categorical; SPQ-BR, Schizotypal Personality Questionnaire; CI, Confidence interval; AP-P, Autistic people with psychosis; NC, Neurotypical controls; AQ, Autism quotient; ALS-18, Affective lability scale-18; ERQ-9, Emotional regulation questionnaire-9; QCAE, Questionnaire of cognitive and affective lability; CR, Cognitive reappraisal. *‘AP-NP’ group taken as reference level. ^†^‘Man’ taken as reference level.

## Discussion

Our study set out to examine whether there was a role for emotional factors to explain existing known relationships between ASD traits and schizotypal traits. In particular, we were interested in the role of emotion regulation, affective lability, and perspective taking/empathy in understanding the relationship between these constructs. Our results replicated those found in previous research in terms of the relationship between AP and NC groups on variables such as schizotypal traits, autistic traits, and empathy. We tested four novel hypotheses, finding that affective processes do appear to contribute to the model of interaction between schizotypal and autistic traits, but our results were not fully conclusive. Taking each hypothesis in turn:

### H1. AP-P Will Use Less Helpful Strategies and Report More Affective Lability Than AP-NP

While in the uncorrected analysis the AP-P group reported significantly greater affective lability, particularly shifts between elation and depression, this difference was not found in the adjusted model. There was no effect found of group on emotion regulation strategy usage, either helpful or unhelpful, and the results provide no corroboration for this hypothesis. However, there is significant gender imbalance between groups, with the AP-P group having proportionally fewer women than the AP-NP group, which may have impacted our ability to identify differences even having controlled for gender. Female AP are known to present differently to male AP in multiple ways (49, 50), and it is unknown how they might differ in their emotion processing.

### H2. AP-P Will Be Better at Perspective Taking Than AP-NP

We found no evidence to support this hypothesis. While AP in general reported worse perspective taking in our study than NC, there was no significant difference between AP groups. This combined with the results of H3, suggest that the psychotic experiences of AP-P are not linked with high levels of empathizing after the onset and resolution of their acute episodes of mental ill-health. We cannot say whether their self-reported empathizing skills would have been at the time of their illness. It is also possible that our study was under-powered to detect differences, as previous research has shown differences between AP-P and AP-NP in empathizing, using a different measure (26). Finally, it is possible that differences might have been found in different types of empathy, and that we simply selected a type of empathy which does not have significant links to psychosis.

### H3. Schizotypal Traits Will Be Higher in AP-P Compared With AP-NP

While this was not true for overall schizotypal traits, it was supported for Positive Schizotypy. Both autistic groups had higher mean levels of schizotypy across the three subscales (Positive, Negative, and Disorganized) than NC, although not all of these comparisons were statistically significant.

### H4. Emotion Regulation Difficulties and Affective Lability Will Be Associated With Higher Schizotypal Scores Across Participant Groups

The premise of this hypothesis is that emotional processes are involved with or impacted by schizotypy, regardless of other factors. This hypothesis was supported. Affective lability has a significant and positive association with overall schizotypy (as one increases, so does the other); further, this is also the strongest association with schizotypy among the variables considered here (r = 0.5, a large effect). This suggests an important relationship between these constructs. Exploratory analysis of the subscales of the ALS-18 suggests that different affective processes may be related to different schizotypal traits. For example, we found that switches between anxiety and depression (ALS-18 anxiety/depression) were more strongly associated with Positive and Negative schizotypy. Switches between depression and elation associated more with Disorganized schizotypy. We also found that, as predicted, Emotional Suppression is significantly associated with greater overall schizotypy, and specifically Positive and Negative schizotypy.

It is important to note that the relationship between emotional processes and negative schizotypy is complex and appears to be affected by the presence of an ASD diagnosis. The two group by measure interactions that we found in the analyses both related to negative schizotypy, and in both cases, the AP groups showed a different relationship between affective lability, emotion regulation, and negative schizotypy, when compared to NC. This suggests that there may be something fundamentally different in the way that AP report experiencing and managing anger. It is also interesting to consider how AP, who show more negative schizotypal traits, report better use of an emotion regulation strategy that relies on logically appraising the situation (Cognitive Reappraisal). The finding may indicate a specific relevance of or different understanding of the descriptions of Cognitive Reappraisal between AP and NC, for example. This requires further investigation.

### Limitations

Potentially impacting on generalizability, this study’s sample sizes were small. This was partially a by-product of selecting a rare dual diagnostic group such as AP-P, and also the nature of the study as exploratory investigation of a novel hypothesis. The sample size particularly impacts on the statistical power to detect interactions. Conclusions, particularly based on interactions, should be interpreted with caution given the small sample size. It may be helpful to consider our results as preliminary in light of these limitations, and further research is clearly required to confirm and expand on them.

Recruitment methods varied between groups, potentially introducing bias: for example, mental status relating to autism and psychosis were retrospectively determined, which may produce bias and impact on the results of the study. Additionally, further screening of individuals may have been useful: AP-NP and NC groups were not screened, and so may have mis-represented their diagnostic status. Psychopathology screening would have helped rule out other confounding relationships. However, additional screening would have added to participant burden and so may have not been as useful as hoped as we are not aware of evidence to suggest research participants have been found to under-represent their mental health status.

Given the limitations of the small sample and limited screening, but the interesting relationships highlighted, future research replicating these results would be valuable. Increased screening of participants to confirm group diagnoses and rule out general psychopathology would help reduce bias from unmeasured confounders. Controlling for additional demographic variables may also further reduce bias. Other designs—such as matching—might also be considered.

### Conclusions

As found in previous research, there appears to be a complex relationship between negative schizotypy, disorganized schizotypy, and autistic traits, and our results have suggested that these traits correlate with emotional processing differences. Future studies would benefit from comparing AP-P to other populations with psychosis to further understanding in this area.

It seems plausible to us that emotional processes, particularly affective lability, add to the model of relationship between autistic and schizotypal constructs. To our knowledge, these factors have not previously been considered in this research field. Lability involving anxious emotional states is associated with positive and negative schizotypy, while lability involving elated emotional states is associated with disorganized schizotypy, suggesting that different emotional experiences may give rise to or be caused by different patterns of thought or behavior. AP as a whole in our study reported significantly higher negative schizotypal traits than NC, replicating previous findings. However, this difference was complicated by interactions with euthymia/anger lability and use of cognitive reappraisal as an emotion regulation strategy, which require further research to replicate and explore further.

Clinically, we would hypothesize that individuals with emotion regulation difficulties and affective lability are likely to be those with either underlying neurobiological differences and/or histories of traumatic experiences such as difficulties during pregnancy/birth [e.g. (51)], insecure early attachments [e.g. (52, 53)], or traumatic events during childhood/adolescence [e.g (54, 55)] which affect neurodevelopmental trajectories. We posit that this would therefore be a potential relevant factor in the development of schizotypal traits that should be further investigated. While differences in attachment have been found between autistic and non-autistic children, these differences seem to be mediated by cognitive ability and level of autistic traits (56), meaning this may not be a risk factor that is greater than in the general population. Little is known about the childhood experiences of AP, including pregnancy and birth issues, and this is an area that would benefit from future study.

We believe that our finding could be considered a development of the stress-vulnerability theory of psychosis (57), and that AP might be particularly at risk due to a combination of neurobiology and life experiences influencing the development of emotion regulation difficulties. In particular, Ford and Crewther’s (16) proposal of a social rigidity factor could be representing the same processes at work in emotional suppression—primarily avoidance and control, as contrasted with openness and flexibility. Understanding the impact of something like social rigidity or other stressors would be key to supporting a program of prevention/strengthening of emotional regulation skills in the at-risk population of teenagers/young AP [see (58) for a possible model]. We believe this is an exciting and transdiagnostic direction for understanding in this field to take, which will ultimately benefit patients through identification of treatment targets and risk markers.

## Data Availability Statement

The raw data supporting the conclusions of this article will be made available by the authors, without undue reservation.

## Ethics Statement

The study involved human participants and was reviewed and approved by North of Scotland NHS Research Ethics Committee. All patients/participants provided informed consent to participate in this study, either written or electronic.

## Author Contributions

FL led design of the study, with contributions from AW, SW, RR, and KC. FL gathered the data. AW conducted data analysis in collaboration with FL. All authors contributed to the article and approved the submitted version.

## Conflict of Interest

The authors declare that the research was conducted in the absence of any commercial or financial relationships that could be construed as a potential conflict of interest.

## Footnotes

1 Identity first language is used throughout this paper. For readers unfamiliar with this concept who wish to learn more about it, we would refer you to https://www.identityfirstautistic.org/ for an introduction.
