# Supplementary material for: Adding a Dimension to the Dichotomy: Affective Processes Are Implicated in the Relationship Between Autistic and Schizotypal Traits
Source: Front Psychiatry. 2020 Jul 24;11:712. doi: 10.3389/fpsyt.2020.00712 (PMC7393992; doi:10.3389/fpsyt.2020.00712)
Supplement: Supplementary file 1 [file Presentation_1.pdf]

Supplementary Material

1 Supplementary Results

1.1 Schizotypy regressions: Controlled relationships with Schizotypy scores (with ALS subscales)

Regression models within 2 of the best AICc for each SPQ-BR scale (Total, Positive, Negative and Disorganised) are shown in Table S7. For two of the models (Positive and Disorganised) the model including no interactions with Group gave the best fitting model (highlighted in yellow in Table S7); additionally, there are no consistently included interactions across the other models considered. For the Total scale, the model with no interactions did *not* have the lowest AICc; however, its fit is only negligibly worse than the best fitting model (999.81 versus 999.56) – given this fit, and the lack of consistent interactions included across the other models, we choose the model with no interactions for reasons of parsimony. For the Negative scale, two interactions are included in the best fitting model, which are also consistently included in the next best fitting model. These are similar to the interactions considered for the negative scale in Table S3. Residual plots indicate no substantial problems with model fit. All VIFs are below four, with the majority below 2.

The fit of the final models is given in Table 4 (the resulting fit with continuous variables standardised is given in Table S8). The amount of variation explained varies from its lowest in disorganised schizotypy (adjusted R^2=0.32) to highest in negative schizotypy (adjusted R^2=0.74). In each of the four regression models, the test of overall regression is significant (p-values<0.001).

For the scales with main effects only models (Total, Positive, and Disorganised) schizotypy differences between groups are minimal. For total schizotypy, there is a significant (p=0.007) difference between NC and AP-P, with AP-P scoring 10.9 units (95% CI: 3.0, 18.6) higher, a small difference (r=0.26) (based on a re-parameterisation of the model in Table 4, not reported). Other pairwise differences between groups on total schizotypy were smaller and non-significant. On positive schizotypy, AP-P score scores significantly higher than the other groups: 6.3 units (95% CI: 1.2, 11.4; p=0.016) higher than AP-NP, a small difference (r=0.23); 10.2 units (95% CI: 5.3, 15.3; p<0.001) higher than NC, a medium difference (r=0.36; from model reparameterization). The positive schizotypy difference between NC and AP-NP was small (r=0.14) and non-significant (p=0.145). Group differences on disorganised schizotypy were all very small (r<0.02) and non-significant (p>0.8).

Except for positive schizotypy, increasing AQ total is significantly associated with increases on the other scales – each unit increase is associated with a: small 0.6 (95% CI: 0.1, 1.0; p=0.017; r=0.23) increase on the Total scale (1 SD AQ gives 0.3 SD Total); medium 0.4 (95% CI: 0.2, 0.6; p<0.001; r=0.40) increase on the Negative scale (1 SD AQ gives 0.5 SD Negative); small 0.2 (95% CI: 0.0, 0.4; p<0.024; r=0.21) increase on the Disorganised scale (1 SD AQ gives 0.4 SD Disorganised).

Except for disorganised schizotypy, increasing ALS-18 Anxiety/Depression is significantly associated with increases on the other scales – each unit increase is associated with a: medium 1.3 (95% CI: 0.5, 2.1; p=0.002; r=0.3) increase on the Total scale (1 SD AQ gives 0.3 SD Total); small 0.7 (95% CI: 0.2, 1.2; p=0.009; r=0.25) increase on the Positive scale (1 SD AQ gives 0.3 SD Positive); medium 0.6 (95% CI: 0.3, 0.8; p<0.001; r=0.36) increase on

the Negative scale (1 SD AQ gives 0.3 SD Negative). On Total and Positive scales, this variable has the largest effect.

ALS-18 Depression/Elation significantly impacts total and disorganised schizotypy – each unit increase in it is associated with a: small 0.6 (95% CI: 0.1, 1.2; p=0.018; r=0.22) increase on the Total scale (1 SD AQ gives 0.2 SD Total); medium 0.5 (95% CI: 0.2, 0.7; p<0.001; r=0.36) increase on the Disorganised scale (1 SD AQ gives 0.4 SD Total). On the disorganised scale, this variable has the largest effect.

For Total, Positive and Disorganised scales, ALS-18 Anger has small (r<0.14) and non-significant impacts.

For the Negative scale, there is an interaction between ALS-18 Anger and participant group, shown in Figure 1, where other variables are averaged across. In the AP-NP group, the ALS-18 Anger score is associated with little change in Negative score: for each point increase on ALS-18 Anger, Negative only decreases by 0.3 (95% CI: -0.6, 0.1; r=0.14) – this is reflected by the blue line in Figure 1, which has the (absolute) shallowest gradient (for AP-NP, 1 SD increase in ALS-18 Anger gives a 0.1 SD decrease on the Negative scale). A steeper, but not significantly different (p=0.304), relationship is seen in the AP-P group: each point increase in ALS-18 anger approximately decreases Negative score by 0.6 [-0.6≈(-0.3)+(-0.3)]. This is depicted in Figure 1 by the pink line in Figure 2, which has a steeper gradient than the blue line (for AP-P, 1 SD increase in ALS-18 approximately gives a 0.2 SD decrease in Negative score). In the NC group, the relationship between ALS-18 Anger and the Negative scale significantly (p=0.006) reverses sign: each point increase in ALS-18 Anger is associated with an approximate *increase* of 0.4 (0.4≈-0.3+0.7) in Negative scale score – giving the green line in Figure 2 with a positive gradient (for NC, 1 SD increase in ALS-18 Anger gives an 0.2 SD increase in Negative).

ERQ-9 Cognitive Reappraisal does not significantly (p>0.08) impact on total, positive or disorganised schizotypy, and the effects are all small (r<0.17). However, there is an interaction between ERQ-9 Cognitive Reappraisal and participant group, shown in Figure S1, where other variables are averaged across. In the AP-NP group, Cognitive Reappraisal score is associated with little change in Negative score: for each point increase on Cognitive Reappraisal, Negative only decreases by 0.02 (95% CI: -0.21, 0.16) – this is reflected by the relatively flat blue line in Figure S1 (for AP-NP, 1 SD increase in Cognitive Reappraisal gives a 0.02 SD decrease in negative schizotypy). The decrease is steeper in the AP-P group, but not significantly (p=0.338) so: each point increase in Cognitive Reappraisal approximately decreases Negative score by 0.22 (-0.22≈-0.02+-.19). This is depicted in Figure S1 by the pink line in Figure 2, which has a steeper gradient than the blue AP-NP line (for AP-P, 1 SD increase in Cognitive Reappraisal gives a 0.16 SD decrease in Negative score). In the NC group, the relationship between Cognitive Reappraisal and negative schizotypy significantly (p=0.018) reverses sign: each point increase in Cognitive Reappraisal is associated with an approximate *increase* of 0.34 (0.34≈-0.02+0.36) in negative schizotypy – giving the green line in Figure 2 with positive gradient (for NC, 1 SD increase in Cognitive Reappraisal gives an 0.25 SD increase in Negative scale score).

Except for disorganised schizotypy, increasing ERQ-9 Emotion Suppression is significantly associated with increases on the other scales – each unit increase is associated with a: small 0.7 (95% CI: 0.3, 1.2; p=0.002; r=0.28) increase on the Total scale (1 SD Emotion Suppression gives 0.2 SD Total); small 0.3 (95% CI: 0.0, 0.6; p=0.046; r=0.19) increase on the Positive scale (1 SD Emotion Suppression gives 0.2 SD Positive); large 0.6 (95% CI: 0.4,

0.7; $p<0.001$; $r=0.54$) increase on the Negative scale (1 SD Emotion Suppression gives 0.4 SD Negative). The impact of Emotion Suppression on the Negative scale is the largest on that and all of the schizotypy scales.

There were no significant relationships between the SPQ-BR subscales and: gender; age; and QCAE Perspective Taking respectively. Additionally, all the non-significant relationships have small effects ($r<0.09$).

1.2 Supplementary Tables

Table S1: *Non-parametric descriptive statistics*. Medians, interquartile ranges (IQRs) by participant group and comparisons between (i) AP groups (Fisher's exact test/ Wilcoxon rank sum) and (ii) all three groups (Fisher's exact test/Kruskal-Wallis rank sum test). Bold/italic p-values indicate significant differences at the 5% level.
AP-NP= Autistic people with *no* psychosis. AP-P= Autistic people *with* psychosis. NC= Neurotypical controls. Stats. Comp= Statistical comparison.
SPQ-BR= Schizotypal Personality Questionnaire. ALS-18= Affective lability scale-18. ERQ-9= Emotional regulation questionnaire-9. QCAE= Questionnaire of cognitive and affective lability. W is the Wilcoxon rank sum statistic. H is the Kruskal-Wallis rank sum statistic. There are two separate sets of comparisons: the first compares the AP groups (middle set of columns) and the second compares all three groups (last set of columns).

	AP-NP			AP-P			NC			ASD comparison (AP-NP v AP-P)		All group comparison	
	**Median**	**IQR**		**Median**	**IQR**		**Median**	**IQR**		**Stats comp**	**P-value**	**Stats comp**	**P-value**
N=	59			23			41						
Gender (% Women)	56%			26%			61%			Fisher's	***0.026***	Fisher's	-
Age (years)	44	36	57	32	26	38	31	28	34	W=1015.5	***0.001***	H(2)=30.1	***<0.001***
AQ Total	41	36	45	33	25	39	20	11	24	W=1028.5	***<0.001***	H(2)=61.3	***<0.001***
SPQ-BR Total	75	63	84	72	64	95	53	38	63	W=607	0.464	H(2)=27.5	***<0.001***
SPQ-BR Positive	22	14	27	26	24	38	14	10	18	W=417.5	***0.007***	H(2)=22.6	***<0.001***
SPQ-BR Negative	29	26	33	26	22	33	19	14	26	W=778.5	0.303	H(2)=25.8	***<0.001***
SPQ-BR Disorganised	24	19	28	21	19	25	19	12	22	W=784	0.277	H(2)=12.9	***0.002***
ALS-18 Total	21	8	30	25	23	34	16	9	26	W=493	0.056	H(2)=6.9	***0.032***
ALS-18 Anxiety/Depression	7	2	10	8	7	11	6	1	9	W=519.5	0.100	H(2)=4	0.138
ALS-18 Depression/Elation	8	3	14	11	9	15	8	4	14	W=500.5	0.066	H(2)=4.1	0.130
ALS-18 Anger	2	0	7	5	4	8	2	0	5	W=495	0.056	H(2)=7	***0.030***
ERQ-9 Cognitive Reappraisal	23	19	27	23	15	24	22	19	25	W=818	0.151	H(2)=2	0.360
ERQ-9 Emotion Suppression	17	12	21	18	13	21	15	10	18	W=641	0.702	H(2)=3.7	0.154
QCAE Perspective Taking	20	15	24	24	16	28	30	26	34	W=545	0.169	H(2)=35.9	***<0.001***

Table S2: *Fitted regressions on ALS-18 Total, ERQ-9 and QCAE scales (continuous variables standardized).* Bold/italic p-values indicate significant terms at the 5% level. Grey cells indicate that the corresponding term has not been included in the regression.
Cat.= Categorical. SPQ-BR= Schizotypal Personality Questionnaire. CI= Confidence interval. AP-P= Autistic people *with* psychosis. NC= Neurotypical controls. AQ= Autism quotient. ALS-18= Affective lability scale-18. ERQ-9= Emotional regulation questionnaire-9. QCAE= Questionnaire of cognitive and affective lability.

		Adjusted comparison (standardized) regressions (n=123)																							
		ALS-18 Total (adjusted R^2=0.43)					ERQ-9 Cognitive Reappraisal (adjusted R^2=0.08)					ERQ-9 Emotion Suppression (adjusted R^2=0.43)					QCAE Perspective Taking (adjusted R^2=0.67)								
Variable	Cat. level	b	95% CI		P-value	r	b	95% CI		P-value	r	b	95% CI		P-value	r	b	95% CI		P-value	r				
Intercept	-	-0.3	-0.6	0.0	0.567	0.054	0.1	-0.3	0.5	0.090	0.160	0.1	-0.2	0.4	0.343	0.090	0.1	-0.2	0.3	***<0.001***	0.743				
Participant Group*	AP-P	0.3	-0.2	0.7	0.266	0.105	-0.3	-0.9	0.3	0.289	0.101	0.1	-0.3	0.6	0.626	0.046	-0.3	-0.7	0.0	0.086	0.162				
	NC	0.5	0.1	1.0	***0.020***	0.218	-0.1	-0.7	0.5	0.723	0.034	0.4	-0.1	0.9	0.105	0.153	-0.2	-0.5	0.2	0.330	0.092				
Gender†	W	0.1	-0.2	0.4	0.636	0.045	0.0	-0.4	0.4	0.945	0.007	-0.4	-0.7	-0.1	***0.008***	0.247	0.1	-0.1	0.3	0.392	0.081				
Age (years)	-	-0.2	-0.3	0.0	***0.023***	0.213	0.1	-0.1	0.3	0.534	0.059	0.0	-0.1	0.2	0.659	0.042	0.0	-0.1	0.1	0.984	0.002				
AQ Total	-	0.4	0.0	0.7	***0.025***	0.211	0.1	-0.3	0.6	0.484	0.067	0.1	-0.3	0.4	0.683	0.039	-0.8	-1.0	-0.6	***<0.001***	0.569				
SPQ-BR Positive	-	0.4	0.2	0.6	***<0.001***	0.406	0.2	-0.1	0.4	0.122	0.146	0.1	-0.1	0.3	0.411	0.078	0.1	-0.1	0.2	0.243	0.111				
SPQ-BR Negative	-	0.0	-0.2	0.3	0.826	0.021	0.0	-0.3	0.3	0.953	0.006	0.7	0.5	0.9	***<0.001***	0.503	-0.2	-0.3	0.0	0.122	0.146				
SPQ-BR Disorganised	-	0.2	0.0	0.4	***0.012***	0.236	0.1	-0.2	0.3	0.632	0.045	-0.2	-0.3	0.0	***0.039***	0.195	0.0	-0.2	0.1	0.508	0.063				
ALS-18 Total	-						-0.3	-0.5	0.0	***0.022***	0.215	0.0	-0.2	0.2	0.894	0.013	0.1	-0.1	0.2	0.354	0.088				
ERQ-9 Cognitive Reappraisal	-											0.0	-0.2	0.1	0.580	0.053	0.1	0.0	0.2	***0.017***	0.224				
ERQ-9 Emotion Suppression	-																0.0	-0.1	0.2	0.554	0.056				

Table S3: Fits of schizotypy regression (with ALS-18 Total) models within 2 of the lowest AICc model and the Interactions with participant group (AP-NP, AP-P and NC) included. A lower AICc indicates a better fitting model. A grey cell indicates where a term has not been fitted in the corresponding regression model. The highlighted row indicates the corresponding reported model in the main paper (eg see Table 3). AICc=Second-order Akaike information criterion. AP-NP= Autistic people with *no* psychosis. AP-P= Autistic people *with* psychosis. NC= Neurotypical controls. AQ= Autism quotient. ALS-18= Affective lability scale-18. ERQ-9= Emotional regulation questionnaire-9. QCAE= Questionnaire of cognitive and affective lability. SPQ-BR= Schizotypal Personality Questionnaire.

		Interactions with participant group (AP-NP, AP-P, and NC)						
**Outcome**	**AICc**	Gender	Age	AQ Total	ALS-18 total	ERQ-9 Cognition Reappraisal	ERQ-9 Emotion Suppression	QCAE Perspective Taking
SPQ-BR Total	999.02	-	-	-	-	-	-	-
	999.17	-	-	-	-	Y	-	-
	999.63	-	-	-	-	-	Y	-
	999.84	Y	-	-	-	-	-	-
	999.85	-	-	-	-	Y	Y	-
	1000.35	Y	Y	-	-	-	-	-
	1000.66	Y	-	-	-	Y	-	-
	1000.70	-	-	-	Y	Y	-	-
	1000.90	-	-	-	-	-	-	Y
SPQ-BR Positive	889.62	-	-	-	-	-	-	-
	890.01	-	-	-	-	-	-	Y
	891.10	-	-	Y	-	-	-	-
	891.17	-	-	-	-	-	Y	-
SPQ-BR Negative	764.38	-	-	-	Y	Y	-	-
	765.00	-	-	-	-	Y	Y	-
	765.03	-	-	-	-	-	Y	-
	765.25	-	-	-	-	Y	-	-
	765.52	-	-	-	-	-	-	-
	765.80	-	-	-	Y	Y	Y	-
SPQ-BR Disorganised	787.76	-	-	-	-	-	-	-
	788.35	Y	-	-	-	-	-	-
	788.45	Y	Y	-	-	-	-	-
	789.65	-	-	-	-	Y	-	-

Table S4: *Fitted Schizotypal regressions (continuous variables standardized)*. Bold/italic p-values indicate significant terms at the 5% level. Cat.= Category. SPQ-BR= Schizotypal Personality Questionnaire. CI= Confidence interval. AP-P= Autistic people *with* psychosis. NC= Neurotypical controls. AQ= Autism quotient. ALS-18= Affective lability scale-18. ERQ-9= Emotional regulation questionnaire-9. QCAE= Questionnaire of cognitive and affective lability. *'AP-NP' group taken as reference level. †'Man' taken as reference level.

		SPQ-BR (standardized) regressions (n=123)																			
		Total (adjusted R^2=0.61)					Positive (adjusted R^2=0.41)					Negative (adjusted R^2=0.68)					Disorganised (adjusted R^2=0.28)				
**Variable**	**Cat. level**	**b**	**95% CI**		**P-value**	**r**	**b**	**95% CI**		**P-value**	**r**	**b**	**95% CI**		**P-value**	**r**	**b**	**95% CI**		**P-value**	**r**
Intercept	-	0.0	-0.2	0.3	0.714	0.035	0.0	-0.3	0.3	0.910	0.011	0.0	-0.2	0.2	0.988	0.001	0.1	-0.2	0.5	0.478	0.067
Participant Group*	AP-P	0.3	-0.1	0.7	0.122	0.145	0.6	0.1	1.1	***0.010***	0.240	0.0	-0.3	0.4	0.895	0.012	-0.1	-0.6	0.4	0.673	0.040
	NC	-0.2	-0.6	0.2	0.265	0.105	-0.3	-0.8	0.2	0.178	0.126	0.0	-0.4	0.3	0.946	0.006	-0.1	-0.7	0.4	0.595	0.050
Gender†	Woman	-0.1	-0.3	0.2	0.672	0.040	0.0	-0.3	0.3	0.790	0.025	0.0	-0.2	0.2	0.979	0.002	-0.1	-0.4	0.2	0.535	0.058
Age (years)	-	0.0	-0.1	0.2	0.545	0.057	0.1	-0.1	0.2	0.356	0.087	0.0	-0.1	0.1	0.740	0.031	0.0	-0.2	0.2	0.740	0.031
AQTotal	-	0.3	0.1	0.6	***0.018***	0.221	0.1	-0.3	0.4	0.728	0.033	0.5	0.2	0.7	***<0.001***	0.345	0.3	-0.1	0.6	0.115	0.148
ALS-18 total	-	0.4	0.3	0.5	***<0.001***	0.505	0.5	0.3	0.6	***<0.001***	0.484	0.1	0.0	0.2	0.058	0.177	0.4	0.2	0.5	***<0.001***	0.354
ERQ-9 Cognitive Reappraisal	-	0.1	0.0	0.2	0.118	0.147	0.1	0.0	0.3	0.090	0.159	0.0	-0.1	0.1	0.706	0.036	0.1	-0.1	0.2	0.432	0.074
ERQ-9 Emotion Suppression	-	0.2	0.1	0.3	***0.002***	0.291	0.2	0.0	0.3	***0.045***	0.187	0.4	0.3	0.5	***<0.001***	0.517	-0.1	-0.3	0.1	0.198	0.121
QCAE Perspective Taking	-	0.0	-0.2	0.2	0.691	0.037	0.1	-0.2	0.3	0.453	0.071	-0.1	-0.3	0.0	0.143	0.137	-0.1	-0.4	0.2	0.465	0.069

Table S5: *Fitted regressions on ALS-18 Total and its subscales.* Bold/italic p-values indicate significant terms at the 5% level. Grey cells indicate that the corresponding term has not been included in the regression.
Cat.= Categorical. SPQ-BR= Schizotypal Personality Questionnaire. CI= Confidence interval. AP-P= Autistic people *with* psychosis. NC= Neurotypical controls. ALS-18= Affective lability scale-18. ERQ-9= Emotional regulation questionnaire-9. QCAE= Questionnaire of cognitive and affective lability. *'AP-NP' group taken as reference level. †'Man' taken as reference level.

Variable	Cat. level	**Adjusted comparison regressions with ALS-18 and its subscales (n=123)**																			
		**ALS-18 Total (adjusted R^2=0.42)**					**ALS-18 Anxiety/Depression (adjusted R^2=0.58)**					**ALS-18 Depression/Elation (adjusted R^2=0.55)**					**ALS-18 Anger (adjusted R^2=0.41)**				
		**b**	**95% CI**		**P-value**	**r**	**b**	**95% CI**		**P-value**	**r**	**b**	**95% CI**		**P-value**	**r**	**b**	**95% CI**		**P-value**	**r**
Intercept	-	-6.0	-26.7	14.7	0.567	0.054	-3.3	-9.7	3.1	0.309	0.097	7.8	-1.5	17.0	0.099	0.158	-5.4	-12.0	1.3	0.114	0.151
Participant Group*	AP-P	3.4	-2.6	9.3	0.266	0.105	1.1	-0.7	3.0	0.219	0.118	-1.1	-3.8	1.5	0.402	0.080	0.9	-1.0	2.9	0.330	0.093
	NC	7.1	1.1	13.1	***0.020***	0.218	1.7	-0.1	3.6	0.068	0.174	-0.5	-3.3	2.2	0.707	0.036	1.1	-0.8	3.1	0.257	0.108
Gender†	Woman	0.9	-3.0	4.9	0.636	0.045	1.6	0.4	2.8	***0.009***	0.248	-0.2	-2.0	1.6	0.822	0.022	-1.4	-2.7	-0.1	***0.035***	0.201
Age (years)	-	-0.2	-0.3	0.0	***0.023***	0.213	0.0	-0.1	0.0	0.896	0.013	-0.1	-0.2	0.0	***0.013***	0.236	0.0	0.0	0.1	0.563	0.055
AQ Total	-	0.4	0.1	0.7	***0.025***	0.211	0.0	-0.1	0.2	0.384	0.083	-0.1	-0.2	0.1	0.322	0.095	0.2	0.1	0.3	***0.004***	0.271
SPQ-BR Positive	-	0.5	0.3	0.7	***<0.001***	0.406	0.1	0.0	0.1	0.057	0.181	0.1	0.0	0.1	0.305	0.098	0.1	0.0	0.1	0.093	0.160
SPQ-BR Negative	-	0.0	-0.3	0.4	0.826	0.021	0.2	0.1	0.3	***0.002***	0.289	-0.2	-0.3	0.0	0.078	0.168	-0.1	-0.2	0.1	0.338	0.092
SPQ-BR Disorganised	-	0.4	0.1	0.7	***0.012***	0.236	0.0	-0.1	0.1	0.521	0.062	0.3	0.1	0.4	***<0.001***	0.359	-0.1	-0.2	0.0	0.260	0.108
ALS-18 Total	-																				
ALS-18 Anxiety/Depression	-											0.6	0.3	0.8	***<0.001***	0.407	0.3	0.1	0.5	***0.006***	0.258
ALS-18 Depression/Elation	-						0.3	0.2	0.4	***<0.001***	0.407						0.2	0.1	0.3	***0.005***	0.263
ALS-18 Anger	-						0.2	0.1	0.4	**0.006**	0.258	0.4	0.1	0.6	***0.005***	0.263					
ERQ-9 Cognitive Reappraisal	-	-0.3	-0.6	0.0	***0.022***	0.215	-0.1	-0.1	0.0	0.174	0.130	-0.1	-0.2	0.1	0.371	0.086	0.0	-0.1	0.1	0.965	0.004
ERQ-9 Emotion suppression	-	0.0	-0.4	0.4	0.894	0.013	-0.1	-0.2	0.0	0.081	0.166	0.2	0.0	0.4	***0.031***	0.205	0.0	-0.2	0.1	0.580	0.053
QCAE Perspective Taking	-	0.2	-0.2	0.6	0.354	0.088	0.1	-0.1	0.2	0.428	0.076	-0.1	-0.3	0.1	0.293	0.101	0.1	0.0	0.2	0.130	0.145

Table S6: *Fitted regressions on ALS-18 Total and its subscales (continuous variables standardized).* Bold/italic p-values indicate significant terms at the 5% level. Grey cells indicate that the corresponding term has not been included in the regression.
Cat.= Categorical. SPQ-BR= Schizotypal Personality Questionnaire. CI= Confidence interval. AP-P= Autistic people *with* psychosis. NC= Neurotypical controls. ALS-18= Affective lability scale-18. ERQ-9= Emotional regulation questionnaire-9. QCAE= Questionnaire of cognitive and affective lability. *'AP-NP' group taken as reference level. †'Man' taken as reference level.v

		Adjusted comparison (standardized) regressions with ALS-18 and its subscales (n=123)																			
		ALS-18 Total (adjusted R^2=0.42)					ALS-18 Anxiety/Depression (adjusted R^2=0.58)					ALS-18 Depression/Elation (adjusted R^2=0.55)					ALS-18 Anger (adjusted R^2=0.41)				
**Variable**	**Cat. level**	**b**	**95% CI**		**P-value**	**r**	**b**	**95% CI**		**P-value**	**r**	**b**	**95% CI**		**P-value**	**r**	**b**	**95% CI**		**P-value**	**r**
Intercept	-	-0.3	-0.6	0.0	0.567	0.054	-0.4	-0.6	-0.1	0.309	0.097	0.1	-0.2	0.4	0.099	0.158	0.0	-0.3	0.4	0.114	0.151
Participant Group*	AP-P	0.3	-0.2	0.7	0.266	0.105	0.2	-0.1	0.6	0.219	0.118	-0.2	-0.6	0.2	0.402	0.080	0.2	-0.2	0.7	0.330	0.093
	NC	0.5	0.1	1.0	***0.020***	0.218	0.4	0.0	0.8	0.068	0.174	-0.1	-0.5	0.3	0.707	0.036	0.3	-0.2	0.8	0.257	0.108
Gender†	Woman	0.1	-0.2	0.4	0.636	0.045	0.4	0.1	0.6	***0.009***	0.248	0.0	-0.3	0.2	0.822	0.022	-0.3	-0.7	0.0	***0.035***	0.201
Age (years)	-	-0.2	-0.3	0.0	***0.023***	0.213	0.0	-0.2	0.1	0.896	0.013	-0.2	-0.3	0.0	***0.013***	0.236	0.0	-0.1	0.2	0.563	0.055
AQ Total	-	0.4	0.0	0.7	***0.025***	0.211	0.1	-0.2	0.4	0.384	0.083	-0.2	-0.5	0.2	0.322	0.095	0.5	0.2	0.8	***0.004***	0.271
SPQ-BR Positive	-	0.4	0.2	0.6	***<0.001***	0.406	0.2	0.0	0.3	0.057	0.181	0.1	-0.1	0.3	0.305	0.098	0.2	0.0	0.4	0.093	0.160
SPQ-BR Negative	-	0.0	-0.2	0.3	0.826	0.021	0.4	0.1	0.6	***0.002***	0.289	-0.2	-0.4	0.0	0.078	0.168	-0.1	-0.4	0.1	0.338	0.092
SPQ-BR Disorganised	-	0.2	0.0	0.4	***0.012***	0.236	0.0	-0.2	0.1	0.521	0.062	0.3	0.2	0.5	***<0.001***	0.359	-0.1	-0.3	0.1	0.260	0.108
ALS-18 Total	-																				
ALS-18 Anxiety/Depression	-											0.4	0.2	0.6	***<0.001***	0.407	0.3	0.1	0.5	***0.006***	0.258
ALS-18 Depression/Elation	-						0.4	0.2	0.6	***<0.001***	0.407						0.3	0.1	0.5	***0.005***	0.263
ALS-18 Anger	-						0.2	0.1	0.4	**0.006**	0.258	0.2	0.1	0.4	***0.005***	0.263					
ERQ-9 Cognitive Reappraisal	-	-0.2	-0.3	0.0	***0.022***	0.215	-0.1	-0.2	0.0	0.174	0.130	-0.1	-0.2	0.1	0.371	0.086	0.0	-0.2	0.1	0.965	0.004
ERQ-9 Emotion suppression	-	0.0	-0.2	0.2	0.894	0.013	-0.1	-0.3	0.0	0.081	0.166				***0.031***	0.205	-0.1	-0.2	0.1	0.580	0.053
QCAE Perspective Taking	-	0.1	-0.1	0.4	0.354	0.088	0.1	-0.1	0.3	0.428	0.076				0.293	0.101	0.2	-0.1	0.4	0.130	0.145

Table S7: Fits of schizotypy regression models (with ALS-18 subscales) within 2 of the lowest AICc model and the Interactions with participant group (AP-NP, AP-P and NC) included. A lower AICc indicates a better fitting model. A grey cell indicates where a term has not been fitted in the corresponding regression model. The highlighted row indicates the corresponding reported model in the main paper (eg see Table 3). AICc=Second-order Akaike information criterion. AP-NP= Autistic people with *no* psychosis. AP-P= Autistic people *with* psychosis. NC= Neurotypical controls. AQ= Autism quotient. ALS-18= Affective lability scale-18. ERQ-9= Emotional regulation questionnaire-9. QCAE= Questionnaire of cognitive and affective lability. SPQ-BR= Schizotypal Personality Questionnaire.

		**Interactions with participant group (AP-NP, AP-P, and NC)**								
**Outcome**	**AICc**	Gender	Age	AQ Total	ALS-18 Anxiety/Depression	ALS-18 Depression/Elation	ALS-18 Anger	ERQ-9 Cognition Reappraisal	ERQ-9 Emotion Suppression	QCAE Perspective Taking
SPQ-BR Total	999.56	-	-	-	-	-	-	Y	-	-
	999.81	-	-	-	-	-	-	-	-	-
	999.81	-	-	-	-	-	-	Y	Y	-
	1000.07	-	-	-	-	-	-	-	Y	-
	1000.88	-	-	-	-	Y	-	Y	-	-
	1001.52	Y	-	-	-	-	-	-	-	-
SPQ-BR Positive	893.24	-	-	-	-	-	-	-	-	-
	894.18	-	-	-	-	-	-	-	-	Y
	894.90	-	-	-	-	-	-	-	Y	-
SPQ-BR Negative	749.34	-	-	-	-	-	Y	Y	-	-
	750.48	-	-	-	-	-	Y	Y	Y	-
SPQ-BR Disorganised	783.61	-	-	-	-	-	-	-	-	-
	785.54	Y	-	-	-	-	-	-	-	-

Table S8: *Fitted Schizotypal regressions with ALS-18 subscales (continuous variables standardized)*. Bold/italic p-values indicate significant terms at the 5% level. Grey cells indicate that the corresponding term has not been fitted in the model. Cat.= Categorical. SPQ-BR= Schizotypal Personality Questionnaire. CI= Confidence interval. AP-P= Autistic people *with* psychosis. NC= Neurotypical controls. AQ= Autism quotient. ALS-18= Affective lability scale-18. ERQ-9= Emotional regulation questionnaire-9. QCAE= Questionnaire of cognitive and affective lability. CR= Cognitive reappraisal. *'AP-NP' group taken as reference level. †'Man' taken as reference level.

		SPQ-BR regressions (with ALS-18 subscales) (n=123)																			
		Total (adjusted R^2=0.62)					Positive (adjusted R^2=0.41)					Negative (adjusted R^2=0.74)					Disorganised (adjusted R^2=0.32)				
Variable	Cat. level	b	95% CI		P-value	r	b	95% CI		P-value	r	b	95% CI		P-value	r	b	95% CI		P-value	r
Intercept	-	0.1	-0.2	0.3	0.500	0.064	0.1	-0.3	0.4	0.733	0.032	0.0	-0.2	0.2	0.792	0.026	0.1	-0.3	0.4	0.622	0.047
Participant Group*	AP-P	0.3	-0.1	0.7	0.131	0.143	0.6	0.1	1.0	***0.016***	0.226	0.1	-0.3	0.4	0.666	0.042	0.0	-0.5	0.5	0.978	0.003
	NC	-0.2	-0.6	0.2	0.242	0.111	-0.4	-0.8	0.1	0.145	0.138	0.1	-0.2	0.4	***0.544***	0.059	0.0	-0.6	0.5	0.851	0.018
Gender†	Woman	-0.1	-0.4	0.1	0.348	0.089	-0.1	-0.4	0.2	0.619	0.047	-0.1	-0.3	0.1	0.380	0.085	-0.1	-0.5	0.2	0.441	0.073
Age (years)	-	0.0	-0.1	0.2	0.476	0.068	0.1	-0.1	0.2	0.401	0.080	0.0	-0.1	0.1	0.900	0.012	0.1	-0.1	0.3	0.425	0.076
AQ total	-	0.3	0.1	0.6	***0.017***	0.225	0.0	-0.3	0.4	0.857	0.017	0.5	0.3	0.8	***<0.001***	0.400	0.4	0.1	0.8	***0.024***	0.212
ALS-18 Anxiety/Depression	-	0.3	0.1	0.4	***0.002***	0.295	0.3	0.1	0.5	***0.009***	0.245	0.3	0.1	0.4	***<0.001***	0.364	0.0	-0.2	0.3	0.726	0.033
ALS-18 Depression/Elation	-	0.2	0.0	0.4	***0.018***	0.223	0.2	0.0	0.4	0.107	0.153	-0.1	-0.2	0.0	0.210	0.121	0.4	0.2	0.7	***<0.001***	0.364
ALS-18 Anger	-	0.0	-0.1	0.2	0.919	0.010	0.1	-0.1	0.3	0.184	0.126	-0.1	-0.3	0.0	0.145	0.140	-0.1	-0.3	0.1	0.302	0.098
ERQ-9 Cognitive Reappraisal	-	0.1	0.0	0.2	0.089	0.161	0.1	0.0	0.3	0.082	0.164	0.0	-0.2	0.1	0.794	0.025	0.1	-0.1	0.2	0.392	0.081
ERQ-9 Emotion Suppression	-	0.2	0.1	0.3	***0.002***	0.282	0.2	0.0	0.3	***0.046***	0.188	0.4	0.3	0.5	***<0.001***	0.540	-0.1	-0.3	0.0	0.105	0.153
QCAE Perspective Taking	-	0.0	-0.2	0.2	0.820	0.022	0.1	-0.2	0.3	0.492	0.065	-0.1	-0.2	0.1	0.441	0.075	0.0	-0.3	0.2	0.811	0.023
Participant Group × ALS-18 Anger	AP-P:Anger											-0.1	-0.4	0.1	0.304	0.099					
	NC:Anger											0.3	0.1	0.6	***0.006***	0.263					
Participant Group × ERQ-9 Cognitive Reappraisal	AP-P: ERQ-9 CR											-0.1	-0.4	0.2	0.338	0.093					
	NC: ERQ-9 CR											0.3	0.0	0.5	***0.018***	0.226					

1.3 Supplementary Figures

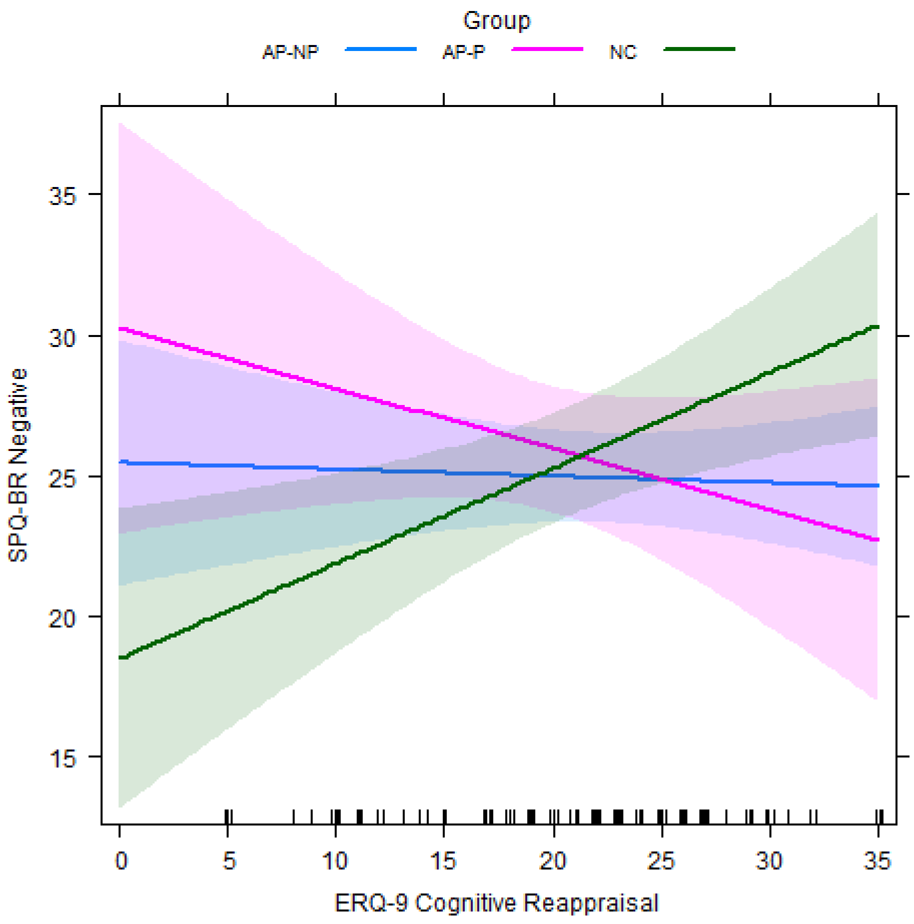

Supplementary Figure 1: Fitted interactions between participant group and ERQ-9 Cognitive Reappraisal in SPQ-BR Negative regression with ALS-18 subscales (see Section 3.2; averaged across other variables). Coloured regions indicate the 95% confidence intervals. AP-NP= Autistic people with no psychosis. AP-P= Autistic people with psychosis. NC= Neurotypical controls. SPQ-BR= Schizotypal Personality Questionnaire. ERQ-9= Emotional regulation questionnaire-9.
